# Characteristics of dementia-friendly hospitals: an integrative review

**DOI:** 10.1186/s12877-022-03103-6

**Published:** 2022-05-31

**Authors:** Christina Manietta, Daniel Purwins, Anneke Reinhard, Christiane Knecht, Martina Roes

**Affiliations:** 1 Deutsches Zentrum für Neurodegenerative Erkrankungen (DZNE), Site Witten, Witten, Germany; 2 Faculty of Health, School of Nursing Science, Witten/Herdecke University, Witten, Germany; 3 FH Münster University of Applied Sciences, Münster, Germany

**Keywords:** Cognitive impairment, Dementia, Acute care, Hospital, Dementia-sensitive

## Abstract

**Background/Objectives:**

Dementia-friendly initiatives are becoming common in society, politics, and research, including health care. Regarding efforts to improve care for patients with dementia in hospitals, the term dementia-friendly hospital is being used increasingly. However, a theoretical understanding of this term and the underlying concept are missing. This integrative review aims to identify current descriptions of dementia-friendly hospitals and to analyze their characteristics.

**Methods:**

An integrative review was conducted. The databases MEDLINE, CINAHL, PsycInfo, Cochrane Library, and additional resources were searched. Two reviewers independently screened publications for inclusion. We extracted data from the included publications and analyzed the descriptions of dementia-friendly hospitals using inductive content analysis in an iterative process.

**Results:**

We identified 4191 records and included 34 publications on 17 descriptions of dementia-friendly hospitals. These were found in the context of practice projects (*n* = 8), recommendations (*n* = 6) and research (*n* = 3). Our analysis resulted in six characteristics of dementia-friendly hospitals. Characteristics related to the patients and their care are *continuity*, *person-centeredness, consideration of phenomena within dementia* and *environment*. Additional characteristics are *valuing relatives* and *knowledge and expertise* within the hospital.

**Conclusion:**

Dementia-friendly hospitals are currently characterized more by healthcare practices and professional dementia experts than by the results of empirical studies. Additionally, the perspective of people with dementia is underrepresented in current descriptions. Accordingly, further research is needed that involves people with dementia in order to develop a theoretical understanding and suitable concepts of dementia-friendly hospitals, since their perspective is essential.

**Supplementary Information:**

The online version contains supplementary material available at 10.1186/s12877-022-03103-6.

## Background

Dementia-friendly initiatives are becoming more and more common in a variety of settings, such as dementia-friendly communities [1], prisons [2], the arts [3], environmental design [4] and healthcare [5] and targeted in national dementia plans [6, 7]. The World Health Organization’s global action plan set a target to establish at least one dementia-friendly initiative in 50% of countries by 2025 [8]. Dementia-friendly initiatives focus on people with dementia and the protection of their human rights, their integration into society and the reduction in stigma [8]. They aim to create a supportive, inclusive and empowering social and physical environment for people with dementia, their families and caregivers [8, 9].

The term “dementia-friendly” is also increasingly used in international publications and national dementia strategies in the context of hospitals [10–12]. Hospitals are in general an unbefitting environment for people with dementia because of functional care, processes, architecture, noise and the presence of strangers [13–15]. Hospitalization is a burden for this patient group and their relatives [13, 14] and is associated with negative experiences for both of them [16–18]. Patients with dementia described their hospital experiences as feeling lost in the environment, being an outsider, boredom, and a lack of intersubjective relationships [18]. Additionally, a hospital stay is often associated with various adverse events and leads to poor outcomes for patients with dementia, such as a longer hospital stay, postoperative complications or moving into a nursing home [13].

To improve the care of patients with dementia in hospitals, various efforts have been made, including the creation of dementia-friendly hospitals (DFHs). However, the term DFH is used heterogeneously, and a theoretical understanding of DFHs as well as the underlying concept are lacking.

Scientific overviews of dementia-friendly discourses in policy documents [19] and dementia-friendly initiatives in research exist [9, 20]; however, there is a lack of literature reviews focusing on DFHs.

To close this research gap, we conducted an integrative review with the objectives (a) to identify current descriptions of DFHs and (b) to analyze the characteristics of DFHs.

## Methods

An integrative review according to Whittemore and Knafl [21] was conducted to identify a broad range of descriptions of DFHs from different types of literature and to analyze them comprehensively. An internal pre-established review protocol guided the process.

### Literature search

We decided to include research and non-research publications based on our review objectives. Accordingly, we conducted a comprehensive literature search to identify both types of publications. For the research publications, a systematic literature search in the databases MEDLINE (via PubMed), CINAHL (via Ebsco), Cochrane Library and PsycInfo (via Ebsco) was performed (latest search in March 2022). We derived the search terms first from our review objectives and then from the results of an initial publication search. We used indexing words and free search terms, which we clustered according to the PICo scheme (population, phenomenon of interest, context) [22]. The search string was developed by the first reviewer (CM) and checked by the second reviewers (DP, MR) using Peer Review of Electronic Search Strategies (PRESS) [23]. (The MEDLINE search string is shown in Additional file 2). Owing to the non-research publications also included, which are mainly gray literature, further search strategies were conducted following Godin et al. [24]. Accordingly, two gray literature databases (OpenGrey, Grey Literature Report), two subject databases (GeroLit, CareLit), three search engines (Google, Google Scholar, Livivo) and targeted websites (e.g., the Alzheimer’s Association) were searched. For this purpose, we created simpler search strings based on the MEDLINE search string or, if this was not applicable, we used a simple combination of the search terms. For a Google search, we conducted a systematic procedure [24] and defined various simple search strings in English and German (Additional file 3). In addition, we performed forward citation tracking for the research publications via Google Scholar and backward citation tracking for all publications included.

### Publication selection

We included research and non-research publications containing descriptions of DFHs. The description had to provide content regarding what constitutes a DFH (e.g., contributions including concepts, several components and/or several characteristics of DFHs). Individual components (e.g., special care units) or dementia-friendly initiatives with a narrowed focus (e.g., dementia-friendly education, dementia-friendly environmental design) were only included if they were part of a concept of a DFH and a publication on the whole concept had been published. The term “dementia-friendly hospital” or similar terms such as “dementia-sensitive hospital” had to be used explicitly. Publications published in German and English from 2010 onwards were included. The detailed inclusion criteria are shown in Table 1.

**Table 1:** Eligibility criteria

Criteria	Definition
*Population*	▪The target population of the phenomenon of interest had to have patients with a diagnosed dementia or symptoms of dementia (e.g., unspecified dementia), who were hospitalized ▪There were no restrictions regarding the type and severity of dementia
*Phenomenon of* *interest*	▪The phenomenon of interest was the DFH. DFHs had to be described in detail, i.e. we included only publications which provide content regarding what constitutes a DFH (e.g., contributions including concepts, several components and/or several characteristics) ▪Individual components (e.g., special care unit) or dementia-friendly initiatives with a narrowed focus (e.g., dementia-friendly education, dementia-friendly environmental design) were only included if they were part of a concept of a dementia-friendly hospital and a publication on the whole concept had been published ▪The term “dementia-friendly hospital” or similar terms such as “dementia-sensitive hospital” had to be used explicitly. The term “dementia capable” was excluded because of its more narrowed focus on the needs of people with dementia [6]
*Context*	▪Included are publications whose phenomenon of interest related to the hospital, regardless of the level of care or ownership of the hospital ▪The focus of the phenomenon had to be on the hospital level and not exclusively on specific areas (e.g., emergency room) or ward (dementia-friendly ward) ▪Excluded were rehabilitation facilities, psychiatric specialty hospitals and facilities that were outsourced by the hospital (e.g., urgent care, outpatient, high-tech home care)
*Types of literature*	▪Any type of research publications (i.e., studies of any design, reviews, qualification theses) were included ▪Non-research publications such as books, recommendations, guidelines, project reports, practice articles and policy documents were considered
*Others*	▪Languages: German and English ▪Year: from 2010

Two reviewers (Reviewer 1: CM, Reviewer 2: DP, MR) independently screened first the titles and abstracts and then the full texts of the potentially relevant publications, against the inclusion criteria (Table 1) using Covidence [25]. The Google search screening process was carried out following Godin et al. [24]. To capture as many of the most relevant hits as possible but also a feasible number to screen, the title and the short description underneath the first 150 hits of each search string were screened by one reviewer (CM). Potentially relevant publications were bookmarked, recorded in an Excel spreadsheet and screened in full text by two reviewers (CM, DP) independently against the inclusion criteria. The search was recorded in an Excel spreadsheet. All conflicts in the screening process were solved together by the screening team (CM, DP, MR).

### Data extraction

We merged several publications of the same study, recommendation or project and counted them as one description of a DFH because they contained essentially the same components of a DFH and differed in their level of detail or included supplementary information (e.g., on project evaluation, participants, funding). Therefore, we grouped the included publications into primary and additional publications. Publications with the more detailed description of a DFH were referred to as primary publications. Publications with less information on the DFH or only containing supplementary information were referred to as additional publications.

We created a standardized form to extract the following information from the included publications: general information on the publications (e.g., author, year, country, publication type, funding), information on the study, recommendation, or practice project (e.g., aim, design/methods, participants, target group) and information on the phenomenon of interest (e.g., term, definition, development, target group, hospital, key components). The data were extracted by one reviewer (CM or AR) and checked by a second reviewer (CM or DP).

### Data evaluation

The aims of our review were to identify and analyze the current descriptions of DFHs, regardless of the publication quality or type. Each description of a DFH was assumed to contribute to the understanding of DFHs. Since the quality of the article was not relevant for our review aims and would not have influenced our publication selection, analysis (e.g., weighting of studies and practice projects) or results, we did not perform a quality assessment of the included publications [21].

### Data analysis

First, we used the extracted data for a detailed illustration of the included descriptions. Then, we analyzed the included descriptions of DFHs using inductive content analysis [26] in an iterative process to identify the characteristics of DFHs. The description of the primary publication was analyzed first and then the analysis was supplemented by the descriptions from the additional publications, if available. The inductive content analysis process was divided into open coding, creating categories and abstraction [26]. First, the descriptions of DFHs were read intensively, notes and headings were made and subcategories were created. Then similar subcategories were grouped into categories and these were grouped depending on their content to create main categories [26]. The analysis was carried out by two reviewers (CM, DP) independently. The categories of the two reviewers were regularly discussed and merged during the analysis process by the screening team (CM, DP, MR) until all descriptions had been analyzed. Abstraction at the level of the main categories was carried out together in the screening team. Finally, the category system was peer-checked, discussed and adapted by the entire review team (CM, DP, AR, CK, MR). For the data analysis, we used MAXQDA 2020.4.1 [27], and for the graphical presentation, SimpleMind [28].

## Results

**Fig. 1:**
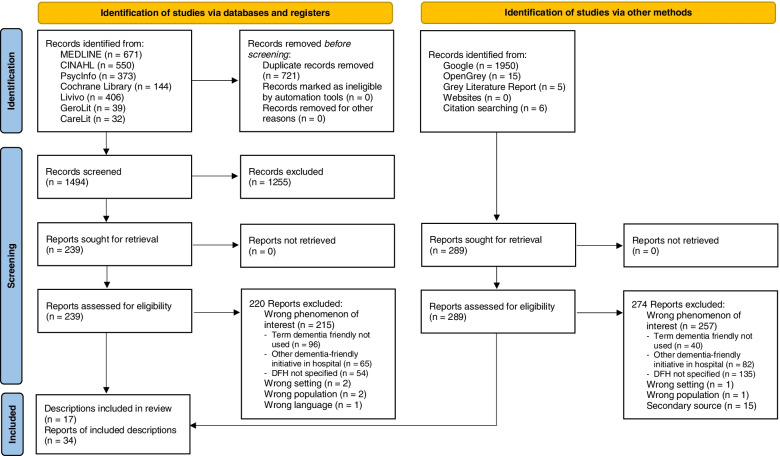
PRISMA 2020 flow diagram [63]

We identified a total of 4191 records via databases and other methods. After deduplication, 3470 titles and abstracts were screened for inclusion. Of these, 528 publications were screened in full text. Most publications were excluded in the full-text screening because the term DFH was used but not specified at all or described in detail (*n* = 189). Finally, we included 34 publications on 17 different descriptions of DFHs (Fig. 1).

### Included descriptions of DFHs

Descriptions of DFHs were mostly found in publications of practice projects (*n* = 8) [29–36], followed by recommendations (*n* = 6) from federal organizations, national alliances, foundations or professional dementia experts [37–42] and research publications (*n* = 3) [43–45]. We included descriptions from Germany (*n* = 12) [29–31, 33–36, 38, 39, 42, 45], Austria (*n* = 2) [37, 41], England (*n* = 1) [40], Denmark (*n* = 1) [43] and Taiwan (*n* = 1) [44].

Most of the descriptions of DFHs reflected the perspective of healthcare professionals or professional experts in the field (*n* = 14) [29–39, 41, 42, 45, 46]. Two of the descriptions included the perspectives of people with dementia and relatives of people with dementia [43, 44], while three of the descriptions included the perspective of representatives of people with dementia (e.g., the Alzheimer’s Association) [43, 46, 47]. Additionally, some descriptions of DFHs used the SPACE principles[48] of the Royal College of Nursing [37, 40, 41, 45] and one the palliative care concept of Silviahemmet [29] as an underlying concept.

The descriptions consisted of several components of DFHs [30, 38, 40, 41, 43–45] or concepts of DFHs [29, 31–37, 39, 42]. Three of these descriptions included an explicit definition of DFHs [39, 41, 45].

A detailed illustration of all included descriptions is provided in Table 2.

**Table 2:** Included descriptions of DFHs

**Publication**	**Research**	**Phenomenon of interest (DFH)**	
**Primary publication:** Schmidt, 2017 [45] **Additional publications:** Lautenschläger et al., 2016 [64] **Country:** Germany **Publication type:** Master thesis, practice article **Funding:** Not reported	**Aim:** To identify central characteristics of a dementia-sensitive hospital and to develop a literature-based instrument to evaluate the dementia-sensitivity of a hospital **Design/Methods:** Literature review, qualitative design (expert interviews, deductive content analysis), quantitative design **Participants:** *Professional dementia experts* (*n* = 6) Professions: nursing, therapists, experts from gerontology, architects	**Used Term:** Dementia-sensitive hospital **Definition:** *“A dementia-sensitive hospital is characterized overall by a processes and strategies based on the needs of people with dementia (PwD). Due to a high level of acquired, communicative competence in the interaction with PwD and a positive attitude of the staff with at least basic dementia training, the PwD and their significant persons are supported during their hospital stay according to their needs. In addition to an early identification of PwD as the prerequisite for an individually tailored treatment and care process, an oriented environment supports a high degree of autonomy and safety”* (Schmidt, 2017 [45], p. 39) **Development:** Based on the literature, expert interviews, the SPACE principles **Target group:** People with dementia	**Key components:** -Staff knowledge and attitude -Cross-sectoral networking -Involvement and support of relatives -Assessment of dementia/other care phenomena -Tailored treatment process and care plan -Environmental design
**Primary publication:** Toubol et al., 2020 [43] **Additional publications:** - **Country:** Denmark **Publication type:** Research article **Funding:** No financial support	**Aim:** To explore and describe stakeholders’ perspectives of a dementia-friendly hospital **Design/Methods:** Qualitative design, focus group interviews, thematic analysis **Participants:** *People with dementia* (*n* = 4): Age group ≥ *65*: *n* = 4 *Relatives* (*n* = 5): Age ≥ 65: *n* = 2, Relationship: *s*pouse (*n* = 4), daughter (*n* = 1) *Hospital staff* (*n* = 4): Age group ≤ 65: *n* = 4, Profession: cleaner (*n* = 1), head of department (*n* = 1), nurse (*n* = 1), physiotherapist (*n* = 1) *Representatives from Alzheimer Association* (*n* = 3): Age group ≥ 65: *n* = 2, Membership background: relative (*n* = 1), relative and nurse (*n* = 1), nurse (*n* = 1)	**Used Term:** Dementia-friendly hospital **Definition:** Not reported **Development:** Results of the qualitative analysis **Target group:** People with dementia in hospitals	**Key components:** Seeing the person behind the dementia diagnosis -Balance of knowledge o About the person oAbout dementia -Facilitating protection oRespectful disclosure of the dementia diagnosis oProtective surroundings oInvolvement of significant others
**Primary publication:** Wu et al., 2019 [44] **Additional publications:** - **Country:** Taiwan **Publication type:** Research article **Funding**: Department of Health, Taipei City Government (105MN14 M); and Healthy Aging Research Center, Chang Gung University (EMRPD1H0361, EMRPD1H0551)	**Aim:** To identify dementia-friendly community indicators from the perspectives of people with dementia and family caregivers **Design/Methods:** Qualitative Design, interviews, content analysis **Participants:** *People with dementia* (*n* = 16): Age: 72.12 (SD 9.38) Dementia level: severe (*n* = 0), moderate (*n* = 5), mild (*n* = 10), mild cognitive impairment (*n* = 1) *Family caregivers* (*n* = 20): Age: 60.15 (SD 10.05) Relationship: spouses (*n* = 9), daughters (*n* = 8), son (*n* = 1), daughter‐in‐law (*n* = 1), sister (*n* = 1) Dementia level (person cared for): mild (*n* = 10), moderate (*n* = 8), severe (*n* = 2)	**Used Term:** Dementia-friendly hospital **Definition:** Not reported **Development:** Results of the qualitative analysis **Target group:** People with dementia in communities	**Key components:** -Provide integrated care -Short waiting times -Staff attitude (friendly and supportive)
**Publication**	**Recommendations**	**Phenomenon of interest (DFH)**	
**Primary publication:** BMFSFJ, 2020 [38] **Additional publications:** BMFSFJ, 2017 [65] **Country:** Germany **Publication type:** Guideline **Funding:** Federal Ministry for Family Affairs, Senior Citizens, Women and Youth	**Aim:** To collect different approaches to create a strategic direction toward a dementia-sensitive hospital **Development:** Based on the experiences of the Local Alliances for People with Dementia and the results of a “dementia and hospital” symposium with contributions from various experts (e.g., research, hospitals, dementia/geriatric associations) *Method:* Not reported **Created by:** Network Local Alliances for People with Dementia in agreement with Federal Ministry for Family Affairs, Senior Citizens, Women and Youth **Created for:** Hospitals in Germany	**Used Term:** Dementia-friendly/dementia-sensitive hospital/structures **Definition:** Not reported **Development:** See development of recommendation **Target group:** Patients with dementia	**Key components** -Staff knowledge -Transitional management -Environmental design -Daily structure and activities -Involvement of volunteers and relatives
**Primary publication:** Horneber et al., 2019 [42] **Additional publications: -** **Country:** Germany **Publication type:** Practice book **Funding:** Not reported	**Aim:** To be a toolbox for practitioners who are involved in creating a dementia-sensitive hospital **Development:** Not reported **Created by:** The chapters were written by different experts (e.g., healthcare management, nursing, medicine, ethics, architecture, geriatrics, theology, technology) **Created for:** Healthcare professionals in hospital	**Used Term:** Dementia-sensitive hospital **Definition:** Not reported **Development:** Not reported **Target group:** Patients with dementia and/or delirium	**Key components** -Environmental design -Sufficient staff and volunteers and expertise -Communication -Admission management -Identification of cognitive impairment -Diagnostic and treatment of dementia/other care phenomena -Discharge management -Corporate culture (spirituality, ethical reflection, protection of patients’ rights)
**Primary publication:** Juraszovich and Rappold, 2017 [41] **Additional publications:** - **Country:** Austria **Publication type:** Guideline **Funding:** Federal Ministry of Health and Women's Affairs and the Federal Ministry of Social Affairs	**Aim:** To support the creation of suitable conditions for dementia-competent hospitals **Development:** Based on the SPACE principles and together with experts *Method:* Expert panel *Participants:* Experts (*n* = 12) from different disciplines and departments in Austria (e.g., hospital management, hospital board, geriatric societies, hospital societies, politics) **Created by:** Austrian National Public Health Institute **Created for:** Highest level of responsibility and decision-makers, managers of all professional groups in the hospital	**Used Term:** Dementia-sensitive hospital, dementia-competent hospital (it seems that the terms were used synonymously) **Definition:** *“Being dementia competent means being attentive to the concerns and needs of people with dementia, responding to their changed lifestyles, pace, perceptions and needs, and adapting and adjusting processes accordingly. This works well when the entire hospital management is behind the issue”* (Juraszovich and Rappold, 2017 [41], p. 1) **Development:** See development of recommendation **Target group:** Patients with dementia	**Key components** -Staff knowledge and sufficient staff -Partnership involving healthcare providers and relatives -Assessment and risk identification -Individual care -Environmental design -Dementia governance
**Primary publication:** Kirchen-Peters and Krupp, 2019 [39] **Additional publications:** Kirchen-Peters and Krupp, 2019 [66] **Country:** Germany **Publication type:** Practice Guideline **Funding:** Robert-Bosch-Stiftung	**Aim:** To systematically collect the experience and knowledge from existing projects and the literature, and to process them into a practical guide for the modular implementation of measures in hospitals **Development:** Based on best practice projects, dementia strategies, literature and interviews *Methods:* Literature search, document analysis, project analysis, interviews/focus group interviews (qualitative content analysis) *Participants:* Professional dementia experts (*n* = 15) with an expertise of care concepts in hospitals for people with cognitive impairments, project stakeholders (*n* = 17) (not further defined) **Created by:** Robert-Bosch-Stiftung, in cooperation with the Institute for Social Research and Social Economy **Created for:** Managers and staff of acute hospitals	**Used Term:** Dementia-sensitive hospital **Definition:** “*Thus, dementia-sensitivity presupposes sensory sensitivity and is intended to help perceive the needs and expectations of people with dementia and integrate these into the design of care structures and processes. Dementia sensitivity serves as a professional basis for the coordinated development of dementia-sensitive structures and processes”* (Kirchen-Peters and Krupp, 2019 [39], p. 22) **Development:** See development of recommendation **Target group:** Patients with dementia	**Key components** -Staff knowledge -Delirium management -Identification and treatment of dementia -Consultation and liaison services -Special care unit -Dementia-sensitive emergency department -Daily structuring and activities -Involvement and support of relatives -Environmental design -Cross-sectoral approaches
**Primary publication:** National Dementia Action Alliance, 2021 [40] **Additional publications:** - **Country:** England **Publication type:** Charter **Funding:** Not reported	**Aim:** To enable hospitals to create a dementia-friendly environment for people with dementia, their families and caregivers in England **Development:** Not reported **Created by:** National Dementia Action Alliance (not further described) **Created for:** Staff and volunteers in hospitals	**Used Term:** Dementia-friendly hospital **Definition:** Not reported **Development:** Based on the SPACE principles. Following recommendations from the Department of Health and Social Care, principal volunteering was added **Target group:** People with dementia, their families and caregivers	**Key components** -Staff knowledge -Partnership with people with dementia, their -relatives/caregivers -Assessments of the needs of people with dementia and their relatives -Person-centered care -Environment -Governance -Volunteering
**Primary publication:** Wallner, 2016 [37] **Additional publications:** - **Country:** Austria **Publication type:** Guideline **Funding:** Not reported	**Aim:** To describe the overall concept of a “dementia-friendly hospital” for a specific hospital **Development:** Not reported **Created by:** Hospital of St. John of God Wien. The Author was a part of the expert panel of B Juraszovich and E Rappold [41] **Created for:** Responsible persons, decision-makers and the management level of the Hospital of St. John of God Wien	**Used Term:** Dementia-friendly hospital **Definition:** Not reported **Development:** Based on the SPACE principles **Target group:** People of dementia	**Key components** -Staff knowledge and sufficient staff -Involvement of relatives -Transitional management -Involvement of volunteers -Working together with external care providers -Information exchange within the hospital -Identification and diagnostics of dementia -Consideration of dementia in care -Environmental design
**Publication**	**Practice projects**	**Phenomenon of interest (DFH)**	
**Primary publication:** Blumenrode, 2018 [35] **Additional publications:** Koch et al., 2019 [47] **Country: Germany** **Publication type:** Practice article, book chapter **Funding** Robert-Bosch-Stiftung	**Aim:** To adapt existing care and treatment services to the needs of patients with dementia or older patients in the emergency department and an orthopedic pilot ward **Created by:** *Hospital:* The clinic Stuttgart (2200 beds) *Project team*: Director and experts (e.g., medicine, nursing, therapists, economic, geriatrics, education, social work, architecture) *Partially involved:* Other stakeholders of the pilot ward, case management, corporate development, the Esslingen University of Applied Sciences, the Alzheimer's Association and volunteers **Scientific evaluation:** Individual components	**Used Term:** Dementia- and age-sensitive hospital **Definition:** Not reported **Development:** Based on existing structures and interventions from international “good practice projects” and recommendations **Target group:** Older patients and/or patients with dementia	**Key components** -Identification and diagnostics of cognitive impairments -Dementia and age-sensitive treatment -Staff knowledge -Dementia experts -Delirium prevention -Daily activities -Environmental design -Person accompanying during surgery -Discharge management
**Primary publication:** Koczy et al., 2017 [36] **Additional publication:** Koczy, 2014 [67] **Country:** Germany **Publication type:** Practice articles **Funding:** Robert-Bosch-Stiftung	**Aim:** To develop and implement a concept for a dementia-sensitive hospital **Created by:** *Hospital:* Robert-Bosch-Hospital Stuttgart (800 beds) **Scientific evaluation:** Not reported	**Used Term:** Dementia-sensitive hospital **Definition:** Not reported **Development:** Not reported **Target group:** Patients with dementia	**Key components** -Special care unit -Identification of cognitive impairments -Staff knowledge -Pathway
**Primary publication:** Malteser, 2021 [29] **Additional publication:** Sottong, 2020 [46], Sottong and Hoffmann, 2014 [51], Malteser and DIP, 2017 [68], Hoffmann, 2015 [69] **Country:** Germany **Publication type:** Poster, conference presentation, practice articles, scientific report **Funding:** *Partly*: Free State of Saxony	**Aim:** To transfer the Swedish Silviahemmet Foundation's “Palliative Care Concept for the Care and Support of People with Dementia” to Germany and to develop a care strategy for Malteser hospitals **Created by:** *Hospitals:* Malteser St. Hildegardis Hospital Cologne, and other Malteser hospitals in Germany Further project: *Hospital:* St. Carolus Hospital Görlitz *Project team*: Different managers from nursing, medicine, house technology, service personnel, administration, functional services, social services and pastoral care *Project advisory board:* Different representatives of Alzheimer associations, research, county and healthcare **Scientific evaluation:** Special care unit, the further project	**Used Term:** Dementia-friendly hospital, dementia-sensitive hospital **Definition:** Not reported **Development:** Based on the palliative care concept of Silviahemmet **Target group:** People with dementia in hospital and their relatives	**Key components** -Special care unit -Daily structure -Environmental design -Staff knowledge -Identification of cognitive impairments -Diagnostic management -Involvement of relatives
**Primary publication:** Motzek et al., 2019 [31] **Additional publication: -** **Country:** Germany **Publication type:** Book chapter **Funding:** *Partly****:*** Robert-Bosch-Stiftung, Emmy-Noether-Program	**Aim:** To develop and implement a dementia sensitive hospital concept **Created by:** *Hospital:* Deaconess Hospital Dresden (220 beds) *Steering group:* persons from the Technische Universität Dresden, Protestant University of Applied Sciences Dresden, the Deaconess Hospital Dresden (the nursing and quality management), external employee *Project team:* nursing, medical, administrative and service staff, and experts (relative and staff education, architecture, patient orientated procedures and concepts) **Scientific evaluation:** Individual components	**Used Term:** Dementia-sensitive hospital **Definition:** Not reported **Development:** By various experts **Target group:** Patients with dementia	**Key components** -Patient-orientated procedures and concepts -Pain and delirium management -Daily structure -Staff knowledge -Environmental design -Support of relatives
**Primary publication:** Poppele et al., 2018 [30] **Additional publications:** Schmitt-Sausen, 2015 [49], Lüdecke, Poppele and Kofahl, 2016 [50], Förster, Kügler and Poppele, 2018 [53], Lüdecke, Peiser and Döhner, 2016 [70], Wunder, 2016 [71] **Country:** Germany **Publication type:** Book chapter, practice articles, scientific report, guideline **Funding:** Robert-Bosch-Stiftung	**Aim:** To record and appropriately care for patients over 65 years of age with cognitive impairment during their hospital stay **Created by:** *Hospital:* Protestant Hospital Alsterdorf Hamburg (293 beds) **Scientific evaluation:** Individual components	**Used Term:** Dementia-sensitive hospital **Definition:** Not reported **Development:** By the hospital with different healthcare professionals (e.g., physicians, nurses, therapists, psychologists) and representatives of the Alzheimer Association **Target group:** Older people (> 65 years) with cognitive impairments, their relatives, medical, nursing, and therapeutic staff	**Key components** -Identification of dementia or delirium -Staff knowledge -Guideline for preserving patient autonomy -Environmental design -Special care unit -Discharge management -Consultation and liaison services
**Primary publication:** Schneider, 2019 [32] **Additional publications:** Fuchs and Lang, 2015 [72] **Country:** Germany **Publication type:** Book chapter, scientific report **Funding:** Earmarked donation and own funds	**Aim:** Organizational development to become a dementia-sensitive hospital **Created by:** *Hospital:* General Hospital Bamberg *Project team:* nursing staff **Scientific evaluation:** Individual components	**Used Term:** Dementia-sensitive hospital **Definition:** Not reported **Development:** Stage model was developed by the nursing director based on nursing theories and models by Orem and Wittneben **Target group:** Patients with cognitive impairments, dementia or delirium	**Key components** -Interdisciplinary treatment and care concepts -Identification and fulfilment of specific care needs -Staff knowledge -Dementia experts -Care counseling service -Additional time resources -Special care units -Qualification mix -Specialized departments for geriatrics -Involvement of pre/post-acute providers
**Primary publication:** Schnetter, 2015 [33] **Additional publications:** - **Country:** Germany **Publication type:** Practice article **Funding:** Not reported	**Aim:** To implement a concept for the care of people with dementia in hospital as a basis for a dementia-sensitive hospital **Created by:** *Hospital:* St. Marien Amberg Hospital **Scientific evaluation:** Not reported	**Used Term:** Dementia-sensitive clinic **Definition:** Not reported **Development:** Not reported **Target group:** People with dementia and their relatives	**Key components** -Geriatric assessment -Involvement of relatives -Environmental design -Special care concepts -Information exchange with external care providers -Staff knowledge
**Primary publication:** Thomas and Schlauß, 2017 [34] **Additional publications:** Klimmer, 2017 [52], Kratz and Diefenbacher, 2019 [54] **Country:** Germany **Publication type:** Practice articles, book chapter **Funding:** *Partly:* Robert-Bosch-Stiftung	**Aim:** To implement a comprehensive treatment and support concept for people with dementia **Created by:** *Hospital:* Queen Elisabeth Herzberge Protestant Hospital (750 beds) **Scientific evaluation:** Pending	**Used Term:** Dementia-sensitive hospital; dementia-friendly hospital **Definition:** Not reported **Development:** Based on evidence of delirium prevention and best practice projects **Target group:** Patients with dementia	**Key components** -Dementia project coordinator -Staff knowledge -Counseling and liaison services -Special support services by trained care-aid staff -Special care concepts -Screening of cognitive impairment -Risk identification -Prevention of perioperative delirium -Support of relatives -Special care unit -Specialized departments for geriatrics -Environmental design -Cross-sectoral network

### Characteristics of DFHs

**Fig. 2:**
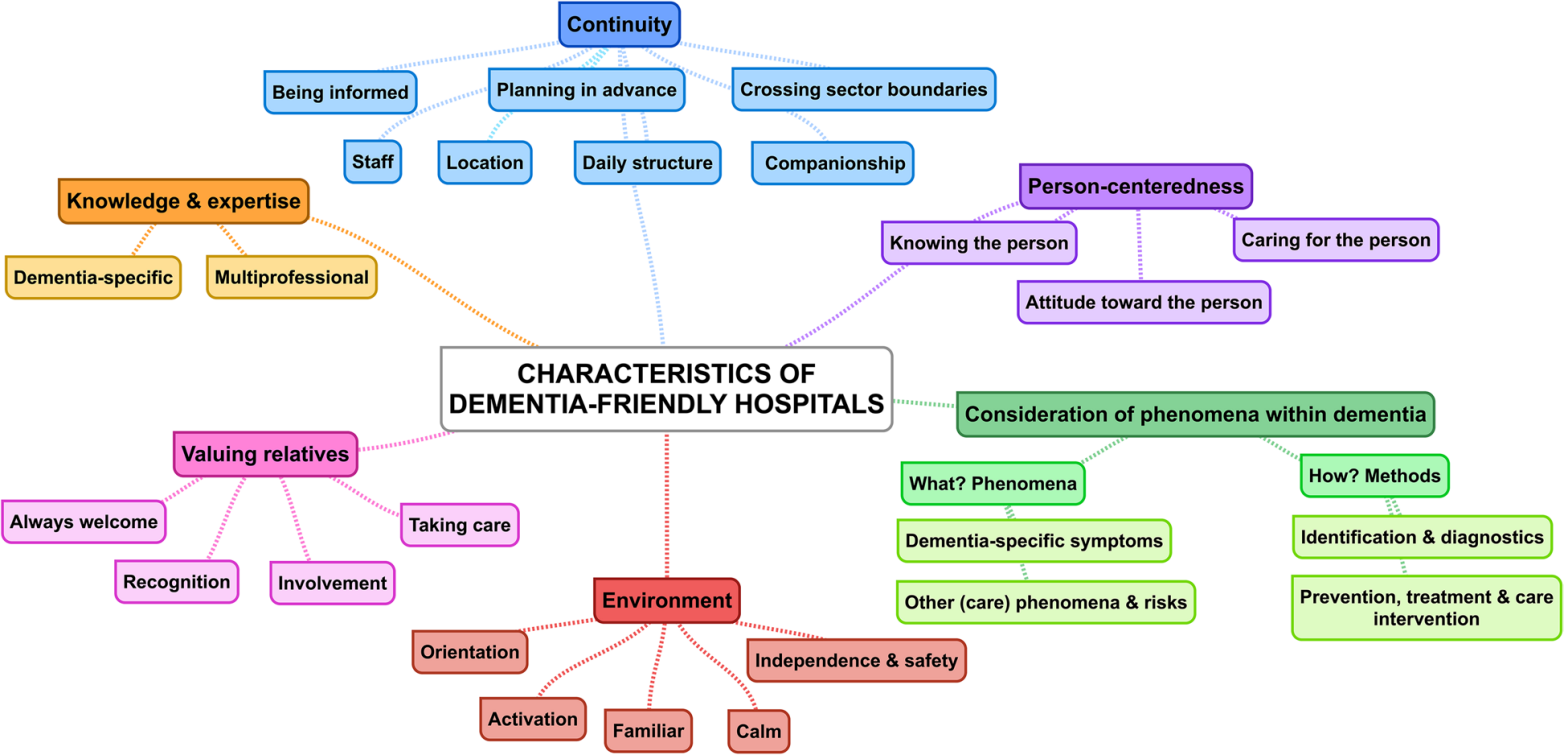
Overview of DFH characteristics

We identified six characteristics of DFHs based on the analysis of the included descriptions: *continuity*, *person-centeredness*, *consideration of phenomena within dementia*, *environment*, *valuing relatives* and *knowledge and expertise* (the full description is presented in Additional file 4). Figure 2 shows an overview of all characteristics.

#### Continuity

Continuity for both the patient with dementia and their care during and after the hospital stay can be created through these aspects: *staff, location, daily structure, companionship, being informed, planning in advance* and *crossing sector boundaries*. The *staff* involved in patient care are characterized by the same people [32, 34, 36, 38, 39, 42, 46, 49], a small group [31, 42] and fixed professional contact persons. These contact persons are described at the level of information and coordination for relatives and external healthcare providers [35, 36, 39, 41, 42, 46] and at the level of a person trusted by patient [31, 34, 39, 42]. Furthermore, to create continuity regarding the *location*, two different strategies are described. First, diagnostics and treatment are carried out in the patient’s room, if possible, so that the patient does not have to leave familiar surroundings [29, 31, 38, 39, 41, 50]. Second, transferring the patient within the hospital or ward is minimized as far as possible [31, 34, 36–40, 42, 45, 50]. Additionally, patients with dementia are supported in structuring their day during the hospital stay, with activities [29, 31, 32, 36–39, 42, 45]. The *daily structure* is tailored to the patient and their daily rhythm. This is also considered in hospital procedures, such as medical examinations, meals or personal hygiene [29, 31, 34, 38, 39, 41, 42, 49–51]. Furthermore, the patient is in the company of relatives or hospital staff who are as familiar with them as possible (*companionship*). Depending on the patient’s needs, there is someone close by on the ward or in the patient’s room to keep them company and to monitor them [31, 34, 37–39, 41, 42, 45, 46]. Additionally, the patient is offered social activities in the company of staff, volunteers or other patients [29–39, 41, 42, 45]. Outside the ward, the patient is escorted by a (familiar) person (e.g., staff, volunteers or relatives) during the admission process [33, 39, 42, 43], to or during examinations [29, 31, 34, 39, 41, 42, 50] and/or surgery [31, 35, 39, 42]. *Being informed* is another aspect of continuity. This means that all professionals involved in patient care have the necessary information to care for the patient during the hospital stay and beyond [38, 39, 41, 42]. It is not always possible for the patient to provide the information themselves, so sharing information with third parties such as internal [34, 36–39, 41, 42] and external healthcare professionals is essential [30, 33, 37–42, 45]. This includes general information about the patient’s condition, symptoms, care or treatment [36, 37, 39, 41, 42], and dementia-specific information, especially the dementia diagnosis [36–39, 42, 43]. *Planning in advance* refers to admission [35, 42, 52], the hospital stay [29, 31, 35, 37, 39–42, 44, 45, 53] and discharge [33, 34, 36–42], which allows the early initiation of interventions that characterize the care of DFHs. For example, during the hospital stay, an early identification of cognitive impairment or dementia is sought to allow early consideration of dementia in the patient’s care [35, 41, 42, 45, 53], and surgical procedures and examinations are planned in advanced so that the patient can avoid waiting times [29, 31, 37, 39, 41, 42, 44, 46]. Additionally, *crossing sector boundaries* by working together [32, 35, 37–40, 42, 44] and networking [34, 38, 39, 42, 45, 46, 53] with other external healthcare providers is targeted to enable coordinated care and to know and develop regional structures.

#### Person-centeredness

Person-centeredness in a DFH is characterized by the following aspects: *knowing the person*, *attitude toward the person* and *caring for the person* with dementia in a person-centered way. *Knowing* or getting to know *the person* with dementia means not only knowing the usual information collected in the hospital, such as medical history, but also the person beyond that. To get to know the person, information such as their behavior [32, 33, 38, 42, 54], their habits [32, 33, 36–39, 42, 50], their preferences [33, 36–42], their biography [31, 32, 38, 39, 41–43, 45, 46, 54] and their relatives and social circumstances [37, 39, 42, 43] are needed. However, there are different views on this kind of information. In the study by Toubol et al. [43], participants with dementia and relatives of people with dementia believe that general information (e.g., current condition, how to fulfill their needs) is sufficient and that there is no need to know everything about the patient’s personal life. However, biographical information is seen by professionals as important for conversations, relationship building and tailored care [32, 39, 41–43]. The *attitude* of staff *toward the person* with dementia is characterized by seeing the patient with dementia as a person and not reducing him or her to the diagnosis [36, 38, 39, 42, 43, 54]. Additionally, this attitude is characterized by empathy [30, 33, 36, 39, 42, 43, 46, 54], respect and appreciation [33, 34, 39, 40, 42–44]. Knowing the person together with the attitude of the staff provide a basis for *caring for the person*, which is characterized by a positive personal relationship [31, 32, 39, 42, 43, 52], the promotion and preservation of the patient’s autonomy and self-determination[30, 38, 40, 42, 43, 52] and tailored care [30, 32, 33, 35, 37–43, 45, 46].

#### Consideration of phenomena within dementia

The focus of care in a DFH is not only on the acute health problem but also on dementia and its consequences for care. Accordingly, dementia itself and dementia-specific symptoms regarding cognition, communication, behavior, and everyday competences are considered in a DFH (*What? Phenomena*) [29–39, 41, 42, 45]. Additionally, other care phenomena and risks − nutrition, delirium, medication or pain − are considered in the context of dementia [30, 31, 33–40, 42, 45, 46]. Therefore, different methods of identification, diagnostics, prevention, treatment and care interventions are described (*How? Methods*). Dementia-specific symptoms, other care phenomena and risks are identified and diagnosed via assessments appropriate for the patient group and medical and/or nursing anamnesis [30, 31, 33–40, 42, 45, 46]. Risks are reduced or avoided by preventive measures such as monitoring (e.g., nutrition protocols), adapting existing processes and treatments (e.g., medication orders, anesthesia) and other interventions (e.g,. hip protectors, finger foods) [30, 31, 33–37, 39–42, 45, 46]. Dementia-specific symptoms and other care phenomena are prevented, treated and taken care of by psychosocial and other non-pharmacological interventions (e.g., aromatherapy, music therapy, cold/heat application for pain) or an adequate pharmacological approach when appropriate (e.g., dementia medication, pain relievers) [31–33, 36, 37, 39, 40, 42, 45, 47, 51, 54].

#### Environment

Another characteristic of a DFH is an environment that supports the patient in terms of *orientation, activation, familiarity, calm, independence and safety*. Different temporal orientation aids (e.g., clocks, calendars, light concepts) [31, 33, 36–39, 41, 42, 45, 46, 52] and local and situational guidance (e.g., color coding and contrasts, signs, information boards) [29–31, 33–43, 45] are described to promote the *orientation* of the patient with dementia in a DFH. Furthermore, the environment provides space for *activation* in terms of social interaction, movement and activities (e.g., seating areas, common room) [29–33, 36–42, 45] and activity items that patients can use to occupy themselves independently (e.g., newspapers, radio, television) [30, 31, 37–39, 42]. A familiar person around the patient [30, 31, 38, 39, 41–43, 52], personal items [30, 37–39, 41, 42, 52], homelike design (e.g., homelike furniture, pictures) [30, 31, 33, 38, 39, 42, 43], and customizable interiors (i.e., interior can be arranged as per individual preferences) [36–38, 41, 42] to create an environment that is *familiar* to the patient. Moreover, an environment is created that contributes to a feeling of *calm* by reducing environmental stimuli (e.g., separate areas, noise reduction) [29–32, 34, 36–43, 45] and providing comforts (e.g., comfortable resting options, temperature adjustments) [39, 42]. Additionally, the environment is characterized by promoting the patient’s *independence* while providing *safety* via various aids (e.g., special beds, automatic lighting systems) [30, 35–39, 41–43, 45, 46] and measures that enable independent access to and use of the premises [31, 33, 37–39, 41, 42, 45, 46] or limit access to hazards and exits [30, 32, 36–39, 42].

#### Valuing relatives

Relatives (i.e., close contacts, both family and non-family members) are valued by DFHs and their staff. Relatives are *always welcome* in the hospital, which means there is a welcoming culture with flexible visiting hours so that relatives can be with the patient at any time [33, 37, 40, 41, 49, 51]. This is also reflected in the hospital’s structures and services, such as rooming-in [30, 31, 33, 36–42, 45, 46], and rooms and interior spaces for relatives (e.g., retreat areas, seating options) [39, 41, 42]. Additionally, relatives are recognized as experts due to their experience with the patient and are valued for this (*recognition*) [30, 32, 33, 35, 38–42, 45, 54] and as partners in the patient’s care [37–40, 42, 46]. Valuing relatives also means enabling them to be involved during the patient’s hospital stay. The *involvement* can occur in different ways through receiving and providing information [30, 32, 33, 36–39, 41, 42, 45], mediation between patient and hospital staff [30, 35, 38, 39, 42, 43, 54], active or passive involvement in care [29–31, 33, 35, 37–43] or decision-making [36, 38–42]. The degree of involvement considers the patient’s and relatives’ wishes, burdens and capabilities [30, 38–43]. Additionally, relatives are also *taken care* of by staff who recognize and consider their needs [30, 38, 40, 42, 46] and offer them tailored support in terms of knowledge about dementia, post-acute care and self-care [30–32, 34–42, 44–46].

#### Knowledge and expertise

In a DFH, both *dementia-specific* and *multiprofessional* knowledge and expertise are available. *Dementia-specific* knowledge and expertise are available at a basic level for all hospital staff [30, 31, 35–43, 45, 46]. In addition, there are dementia or geriatric experts (at ward or hospital level) who can be involved in the care of patients with dementia and support the health care staff [30–42, 45]. Moreover, there is *multiprofessional* knowledge and expertise available for the care of patients with dementia. Therefore, professionals from diverse disciplines are involved in care, and find different ways of working together, such as holding multiprofessional team meetings or case conferences, to bundle their expertise and knowledge [31–42, 45, 46, 49, 53].

#### Characteristics related to the context of the description

All of the six characteristics of DFHs that we identified through our inductive content analysis were found in publications of practice projects, recommendations and research publications. All subcategories of the characteristics are essentially presented in their entirety in the publications of practice projects and recommendations. We identified fewer subcategories for all characteristics in two of the three included research publications [43, 44]. In particular, the characteristic *consideration of phenomena within dementia* were not represented and only individual subcategories of the characteristics *continuity *and* knowledge and expertise* could be identified in these publications. These publications included the perspectives of people with dementia and their relatives, in contrast to the other publications which contained primarily the perspective of healthcare professionals or professional experts (Additional file 5 presents the characteristics in relation to each description).

## Discussion

To our knowledge, our integrative review provides for the first time, a comprehensive overview of current descriptions of DFHs based on a systematic method and the inclusion of different types of literature. Moreover, our analysis clusters and synthesizes the characteristics of DFHs and presents them in a new, comprehensive manner.

The term DFH was frequently used in all types of international literature, including research publications, but was rarely described. Even in the 17 descriptions included, only three provided a definition of the term, and some only described several components of DFHs without explicitly explaining what constitutes a DFH in its entirety. However, the described key components of DFHs did not differ substantially.

We found that the term DFH is more often described in publications of practice projects and recommendations than in research publications. Few of the descriptions were the result of an empirical study. Moreover, most of the included descriptions of DFHs were based solely on the perspective of healthcare professionals or other experts in this field. The perspective of people with dementia was rarely included, and only one description included different perspectives. Our results confirm that the involvement of people with dementia in the development of dementia-friendly initiatives is underrepresented [9]. However, to develop a concept of DFHs that is suitable for patients with dementia, it is essential to involve people with dementia in the development, since they are the only experts on what it feels like to live with dementia and only they can provide this unique perspective on the topic [55–57]. In particular, this would allow a differentiated view and a critical reflection on measures reported in the included descriptions, such as limited access to exits or the collection of personal information, as the study by Toubol et al. [43] partially shows. Nevertheless, it is important to involve different stakeholder groups — people with dementia, their relatives, healthcare professionals and researchers — in the development of DFHs. This approach would lead to suitable concepts in a more differentiated and targeted way, and inappropriate interventions could be reduced.

We identified six characteristics of DFHs: *continuity*, *person-centeredness*, *consideration of phenomena within dementia*, *environment*, *valuing relatives* and *knowledge and expertise*. These characteristics address the needs of patients with dementia, their relatives and healthcare professionals as identified in other studies [15, 16, 58–61]. In particular, person-centeredness is a central topic in other studies that interviewed patients with dementia and their relatives. Aspects, such as being accepted and treated as a valued person, being involved in their own care and conversations, and receiving personalized and tailored care, are described in these studies and seem to be important for this patient group [16, 58, 60, 61]. In contrast to current initiatives for an age-friendly health system such as the 4Ms framework (What Matters, Medication, Mentation and Mobility) [62], which, among other things, considers the mentation of older people, the characteristics of a DFH we identified seem to address the needs of people with dementia more specifically and comprehensively.

In addition to the needs of people with dementia, their relatives and healthcare professionals, the characteristics we identified are also reflected in the goals of dementia-friendly initiatives in general. Accordingly, raising awareness about people with dementia, maintenance of their human rights, care that meets their needs and a supportive environment for them and their relatives [8] seems to be an overriding goal of DFHs. However, these goals can only be achieved by listening to and understanding people with dementia as “experts by experience” [55, 57] and incorporating their perspectives into current healthcare practice and concepts of DFHs.

### Limitations

There are potential limitations of our integrative review that need to be considered. We only included publications in English and German. This may have led to a bias, especially regarding the non-research literature, which is often published in the native languages. We conducted a comprehensive search of databases and used different strategies to identify non-research literature, but we might have missed publications or descriptions on the topic.

In addition, it should be noted that only publications that included a detailed description of DFHs were included; this may have biased the results. This review focused on DFHs; accordingly, only publications with this or similar terms (e.g., “dementia-sensitive”) were considered to specify the understanding of DFHs. Publications without the term were excluded, which might have resulted in similar concepts described in other terms being overlooked.

## Conclusion

Our integrative review provides an overview of current international descriptions of DFHs. Moreover, our analysis provides an initial understanding of DFHs and their characteristics and contribute to a consistent terminology of DFHs in the future. Although our identified characteristics are consistent with the needs of patients with dementia, it is important to keep in mind that these results are mainly derived from descriptions of DFHs based on the perspective of healthcare professionals and professional dementia experts. Accordingly, these characteristics can only be used as orientation for health care practice and research at this stage. However, our results can contribute to raising the awareness of healthcare professionals. Based on our identified characteristics of DFHs, healthcare professionals can reflect on their provision of care, hospital structures and processes and thus provide an impulse to move towards a DFH. For researchers, our results are important because they form an essential basis for further research in this field. Additionally, future research needs to explore synergies and overlaps between DFHs and other approaches such as age-friendly initiatives (e.g., 4Ms framework). This includes exploring whether these initiatives could be supplemented by aspects of DFHs. Based on our review results, future research needs to consider the following: (1) to reflect the characteristics of DFHs with people with dementia, their relatives and healthcare professionals, (2) to operationalize and tailor the characteristics of DFHs together with these target groups, and (3) to implement and evaluate the characteristics of DFHs across hospitals (nationally and internationally) so that country-specific and cross-country comparisons become possible. Since the perspective of people with dementia and their relatives on DFHs was underrepresented in the included descriptions, it is of utmost importance to involve them in a participatory way in the future.

## Supplementary Information

**Additional file 1.** PRISMA checklist.

**Additional file 2.** Search strategy example in Medline.

**Additional file 3.** Google search.

**Additional file 4.** Detailed description of DFH characteristics.

**Additional file 5.** Overview of characteristics in each description.

## Data Availability

All data generated or analyzed during this review are included in this published article and its supplementary information files.

## Abbreviations

DFH: Dementia-friendly hospital
PICo: Population, phenomenon of interest, context
