# Supplementary material for: Characteristics of dementia-friendly hospitals: an integrative review
Source: BMC Geriatr. 2022 May 31;22:468. doi: 10.1186/s12877-022-03103-6 (PMC9158310; doi:10.1186/s12877-022-03103-6)
Supplement: Supplementary file 2 — Additional file 2. Search strategy example in Medline. [file 12877_2022_3103_MOESM2_ESM.docx]

**Additional file 2:** Search strategy example in Medline (via PubMed)

| **Population** | #1 "Dementia"[Mesh]  #2 mild cognitive impairment[TIAB]  #3 Alzheimer*[TIAB]  #4 dement*[TIAB]  #5 OR/1-4 |
| --- | --- |
| **Context** | #6 "Hospitals"[Mesh]  #7 acute care[TIAB]  #8 inpatient[TIAB]  #9 acute setting*[TIAB]  #10 hospital[TIAB]  #11 OR/#6-10  #12 #5 AND #11 |
| **Phenomenon of interest** | #13 sensitiv*[TIAB]  #14 friendly [TIAB]  #15 responsive [TIAB]  #16 awareness [TIAB]  #17 OR/#13-16  #18 #12 AND #17 |
