# Supplementary material for: Characteristics of dementia-friendly hospitals: an integrative review
Source: BMC Geriatr. 2022 May 31;22:468. doi: 10.1186/s12877-022-03103-6 (PMC9158310; doi:10.1186/s12877-022-03103-6)
Supplement: Supplementary file 3 — Additional file 3. Google search. [file 12877_2022_3103_MOESM3_ESM.docx]

**Additional file 3:** Google search

Note: no filter

Search: "All results", first 15 pages (150 hits), representing a total of 1950 screened hits

| # | Search | Search date | Total hits | Hits screened* | New potentially relevant  records† | Records included |
| --- | --- | --- | --- | --- | --- | --- |
| 1 | demenzfreundliches Krankenhaus | 30 Mar 2020 | ~ 13800 | 150 | 48 | 5 |
| 2 | demenzsensibles Krankenhaus | 01 Apr 2020 | ~ 30600 | 150 | 33 | 1 |
| 3 | demenz sensitives Krankenhaus | 15 Apr 2020 | ~ 53400 | 150 | 3 | 0 |
| 4 | demenzfreundliche* Universitätsklinik* | 20 Apr 2020 | ~ 4520 | 150 | 15 | 1 |
| 5 | demenzsensible* Universitätklinik* | 23 Apr 2020 | ~ 8560 | 150 | 23 | 0 |
| 6 | demenz sensitiv* Universitätsklinik* | 25 June 2020 | ~ 38700 | 150 | 0 | 0 |
| 7 | demenzfreundliches Spital | 25 June 2020 | ~ 3620 | 150 | 5 | 2 |
| 8 | demenzsensibles Spital | 25 June 2020 | ~ 14500 | 150 | 45 | 1 |
| 9 | demenz sensitives Spital | 26 June 2020 | ~ 65000 | 150 | 1 | 0 |

| # | Search | Search date | Total hits | Hits screened* | New potentially relevant  records† | Records included |
| --- | --- | --- | --- | --- | --- | --- |
| 10 | dementia friendly hospital* | 21 Aug 2020 | ~ 19400000 | 150 | 60 | 1 |
| 11 | dementia friendly acute care | 25 Sep 2020 | ~ 12500000 | 150 | 35 | 0 |
| 12 | dementia sensitiv* hospital* | 29 Sep 2020 | ~ 50100000 | 150 | 7 | 0 |
| 13 | dementia sensitiv* acute care | 29 Sep 2020 | ~ 75200000 | 150 | 10 | 0 |

* title and short text underneath screened by one reviewer

† full text screened by two reviewers
