# Supplementary material for: Characteristics of dementia-friendly hospitals: an integrative review
Source: BMC Geriatr. 2022 May 31;22:468. doi: 10.1186/s12877-022-03103-6 (PMC9158310; doi:10.1186/s12877-022-03103-6)
Supplement: Supplementary file 4 — Additional file 4. Detailed description of DFH characteristics. [file 12877_2022_3103_MOESM4_ESM.docx]

**Additional file 4:** *Detailed description of DFH characteristics*

| Category/subcategories | | Description |
| --- | --- | --- |
| Category:  Continuity | | Continuity is created for both the patient with dementia and their care during and after the hospital stay**.** Continuity is characterized by staff, location, daily structure, companionship, being informed, planning in advance and crossing sector boundaries. |
| Subcategory:  *Staff* | | Continuity of staff is characterized by same staff, a small group of staff and a permanent professional contact person. This helps to build a relationship with the patient. Furthermore, this provides the feeling of continuity for the patient and supports continuity of their care. |
|  | **Operationalization:** |  |
|  | Same staff | Same staff are involved in the care of the patient and changes in staff are avoided. This refers to the various professional groups (e.g., nursing staff, physicians, housekeeping, volunteers). |
|  | Small group of staff | The number of staff involved in the care is reduced to a minimum. |
|  | Professional contact person | There is a permanent professional contact person for information, coordination and concerns during and after the hospital stay for general practitioners, nursing facilities, relatives, patients; this person can be from the area of case management, nursing, or medicine.  A defined contact person is assigned to the patient. This person is involved in the care of the patient as early as possible (preferably from admission to discharge) to build a relationship. This staff member can be staff from geriatric care, nursing, or a volunteer. |
| Subcategory:  *Location* | | Continuity of the location is characterized by service coming to the patient and avoiding internal transitions. |
|  | **Operationalization:** |  |
|  | Service comes to the patient | Diagnostics and treatments are carried out in the patient’s room. A change of location is only made if the examination is not possible any other way (e.g., due to required equipment such as MRI). |
|  | Avoiding internal transitions | Transferring the patient within the hospital is minimized as far as possible by avoiding emergency room stays and changes of the ward or room, e.g. admitting patients directly to the ward where they will stay during their hospitalization. |
| Subcategory:  *Daily structure* | | Daily structure creates continuity for the patients. They are supported in structuring their day, and the hospital procedures are tailored to their individual daily rhythm and structure. |
|  | **Operationalization:** |  |
|  | Supporting daily structure | Patients are supported in structuring their days (e.g., offering activities, offering defined mealtimes and sequences to rest). |
|  | Tailored daily structure | The daily structure and procedures in the hospital (e.g., examinations, treatments, ward rounds, meals, personal hygiene, bedtimes, daily routine) are flexibly tailored to the daily rhythm of the individual patient. |
| Subcategory:  *Companionship* | | Continuity is created for the patient by the company of people who are as familiar with them as possible. Someone is close to the patient on the ward so that the patient does not feel alone. The patient is also offered activities in the company of others and is escorted outside the ward. |
|  | **Operationalization:** |  |
|  | Being close by | Being close by refers to staying on the ward to keep the patient company but also to keep an eye on the patient (monitoring function). This can be in the form of spatial proximity or presence of a person, depending on the patient’s needs.  Spatial proximity: There is spatial proximity of the nurses’ station to the patient's room, common room, seating area and the patient can see the staff.  Presence of a person: The patient is in the presence of a (familiar) person (e.g., staff, volunteer or relative) hourly up to 24-hours. |
|  | Social activities | The patient is offered activities in the company of others such as eating together with other patients, reading aloud, talking, singing or playing to provide companionship and occupation. These activities are offered as individually or in group sessions. |
|  | Escort | Patients are accompanied by a (familiar) person (e.g., staff, volunteer or relatives) during the admission process (e.g., in the emergency room), to and during examinations and therapy, and/or before, during and after surgery (e.g., recovery room). |
| Subcategory:  *Being informed* | | Continuity for the patient and their care is ensured by all professionals involved have the information about the patient that is necessary for their care during the hospital stay and discharge process as well for the post-acute care phase.  If the patient is unable to provide the information themselves, sharing information with third parties such as relatives and internal and external health professionals is essential.  The information needed to provide care includes general information (e.g., the patient’s condition, symptoms, diseases, care, treatment, medication) and dementia-related information (e.g., dementia diagnosis, behavior). |
|  | **Operationalization:** |  |
|  | External | Collecting and sharing information from and with external health care professionals (e.g., general practitioners, outpatient social services, home care, nursing homes, or pharmacists) especially during admission and discharge processes. Information is collected and shared using documents (e.g., discharge letter, nursing report, medication plan, or joint documentation system) and conversations. |
|  | Internal | Exchange information between health care professionals involved in the patient’s care within the hospital. Exchange of information takes place via documentation, hand over, case conferences and meetings. |
| Subcategory:  *Planning in advance* | | Planning in advance means that the patient’s care is planned in advance related to admission, during the hospital stay and discharge to ensure continuity of care. |
|  | **Operationalization:** |  |
|  | Admission | Planning in advance is provided in the form of preparation for a hospital stay, especially in the case of an elective reason for hospitalization.  For planned admissions, assignment to the expected ward is made in advance, as well as preparatory measures on the ward (e.g., room planning in relation to rooming-in). The assignment of a permanent professional contact person can be planned and could take place during preparations for admission. The presence of relatives during the admission process can be scheduled. Required information and documents are collected in advance of the day of admission, so that the necessary information for the patient’s care is available from the beginning of the hospital stay. For planned operations, preparatory measures take place before admission to shorten the hospital stay.  Information can also be prepared for emergency admissions, e.g., in the form of emergency cards or prepared checklists, which have been previously filled out by relatives or health care professionals. |
|  | During hospital stay | Planning in advance includes initiating interventions that are related to early identification of dementia-related symptoms, decisions for care and processes during the hospital stay.  Early identification of cognitive impairment or dementia-related symptoms is sought, enabling the adjustment of care and treatment at an early stage.  If possible, general medical, nursing and psychosocial decisions are made in advance, such as preparing a living will or initiating legal assistance at an early stage.  To avoid waiting times, procedures that are necessary (e.g., surgery or examinations) are planned in advance. |
|  | Discharge | Early discharge planning is conducted to identify and close gaps in care and to ensure that post-acute care interventions are in place. Post-acute care needs and care preferences are identified early, and planning for post-acute care is initiated and planned at an early stage by multidisciplinary professionals (e.g., case management).  Measures necessary for post-acute care are initiated in advance (e.g., application for support services, counseling and training of patients and relatives, initiation of home adaptations, organization of rehab, short-term care, outpatient care or nursing home, procurement of medications, wound dressings and aids). |
| Subcategory:  *Crossing sector boundaries* | | Crossing sector boundaries is characterized by working together and networking with other health care providers especially pre- and posthospital providers to achieve continuity of care during and after the hospital stay. |
|  | **Operationalization:** |  |
|  | Working together | Working together involves the collaboration of external health care professionals and health service providers related to the patient’s care in general (e.g., with general practitioners, nursing facilities), diagnostics or treatment of dementia (if the hospital has no internal dementia experts) and shared care services (e.g., case management, consulting). |
|  | Networking | Regional networking and collaboration is conducted with external health care services (e.g., general practitioners, nursing services, nursing support centers, voluntary structures, community structures, rehabilitation facilities, rescue services) to know and develop regional structures to provide patients with the best possible post-acute care and to exchange experience about the care of people with dementia and to develop common solutions and interventions. |
| Category:  Person-centeredness | | Person-centeredness is characterized by knowing the person with dementia, the positive attitude exhibited toward the person with dementia and caring for the individual in a person-centered way. |
| Subcategory:  *Knowing the person* | | Knowing or getting to know the person with dementia involves acquiring the usual information collected during the hospital stay (e.g., diagnoses or medical history) but also knowing the person beyond that, for example having information about their behavior, preferences, habits, biography and relatives.  Knowing the person is important since it influences the attitudes toward the person and builds a basis for a person-centered care. |
|  | **Operationalization:** |  |
|  | Behavior | Knowing the behaviors (e.g., agitation, wandering), including information to analyze and interpret behavior and feelings (e.g., worries, fears), to identify stressors and triggers that may lead to behavior that challenges. |
|  | Habits | Knowing the habits of the patient related to nutrition, sleep, daily routine, spiritual, grooming. |
|  | Preferences | Knowing the preferences (including likes and dislikes) related to different care aspects, such as general care, nutrition, further care, end-of-life care, activities, environment. |
|  | Biography | Knowing about the life of the patient, aspects of their personality, moral concepts and values, former profession, religious/spiritual background, key experiences, living situation (e.g., house, residential care, alone). |
|  | Relatives | Knowing about their social network, family status, relatives. |
| Subcategory:  *Attitude toward the person* | | The attitude of the staff toward the person with dementia (and their relatives*) is characterized by "seeing the person" behind the diagnosis, empathy, respect and appreciation*. |
|  | **Operationalization:** |  |
|  | Seeing the person | Seeing a patient with dementia as a person with potential, capabilities, needs and dignity. It also means not to reduce them to their diagnosis of dementia or stigmatize them based on that diagnosis and neglect their abilities.  In this context, labeling a patient (e.g., demented patient, wristbands) or their documents (e.g., color marking) is considered critically (an act of stigmatization vs. a safety consideration). |
|  | Empathy | Empathize with the patient with dementia, by applying an empathic interaction, changing one’s perspective (e.g., placing oneself in the other person’s shoes), setting aside personal values and practicing self-reflection. |
|  | Respect & appreciation | Respectful and appreciative interaction with the patient with dementia and their relatives; especially in the context of communication (e.g., talk and listen to the patient, communication on an equal footing, personally address the patient, include the patient in conversations and do not overlook them, respect their views and take them seriously by practicing tolerance, friendliness, and kindness in adequate communication with no deception or lies). |
| Subcategory:  *Caring for the person* | | Caring for the patient in a person-centered way is characterized by fostering a personal relationship with the patient, respect for and promotion of their autonomy as well as care tailored to the person.  To provide care in a person-centered way, knowing the person and having a positive attitude toward the person are essential prerequisites. |
|  | **Operationalization:** |  |
|  | Relationship | Building an (inter)personal relationship based on the subcategories of attitudes toward the person and knowing the person. This relationship is characterized by body contact, perception, creating interaction, connection to the person and budling trust. |
|  | Autonomy | Professionals deem to respect, promote and preserve patient autonomy and self-determination related to their care, treatment, diagnostic, discharge, and post-acute care. This involves the following efforts: involvement, choice and control in decisions; supporting/enabling the patient with dementia to make their own decisions (e.g., by providing information, clear explanations, easy language); shared/participatory decision-making (patient, relative, health care professionals); respecting patient’s wishes and decisions in the situation or if the patient is unable to articulate them, the determine presumed will (e.g., by living will, observed behavior, relatives speaking on behalf of the patient). |
|  | Tailored care | Care is tailored (e.g., care, activities, treatment) to the person based on the patient's individual needs, preferences, habits and demands (i.e., resources and health status). |
| Category:  Consideration of phenomena within dementia | | Dementia and its consequences (e.g. impact on everyday living) but also other phenomena and risks relevant to the care provided, are considered within the context of dementia. The focus of care is thus not only on the acute health issue and the primary reason for hospitalization but also on dementia. |
| Subcategory:  *What? Phenomena* | | Considering dementia-specific symptoms, as well as particularly other care phenomena and risks in context of dementia during hospitalization. |
|  | **Operationalization:** |  |
|  | Dementia-specific symptoms | Consideration of dementia-specific symptoms such as regarding cognition, communication, sensory abilities, everyday competences, as well as behavior that challenges. |
|  | Other (care) phenomena & risks | Consideration of other care phenomena and risks related to nutrition, medications, pain, mobility, falls, restraints, delirium, depression and end of life in the context of dementia. |
| Subcategory:  *How? Methods* | | The phenomena are considered during the hospital stay (if possible) by identifying, diagnosing, preventing, treating and taking care of them. |
|  | **Operationalization:** |  |
|  | Identification  & diagnostics | Phenomena (other and dementia-specific symptoms) and risks are identified at an early stage (i.e., as part of the admission process or in the course of the hospital stay if phenomena occur) by means of assessment (which are suitable for people with dementia), tests, checklists, anamnesis (medical, nursing), professional observation, specific questions, information from relatives and external health care professionals.  After following the identification of dementia-specific symptoms (e.g., cognitive impairment), differentiated diagnostics with various examinations are initiated and differential diagnostics for depression and delirium are carried out, if necessary (e.g., not with already existing diagnosis). |
|  | Prevention, treatment & care intervention | Risks are reduced or avoided by preventive measures such as monitoring (e.g., nutrition logs), adapting existing processes and treatments (e.g., medications, anesthesia) and providing interventions (e.g., hip protectors, activities, mealtimes together, finger foods). Additionally, dementia-specific symptoms regarding cognition and everyday competences are promoted and maintained by preventive measures (e.g., geriatric rehabilitation, cognitive training).  Phenomena (other and dementia-specific symptoms) are taken care of by psychosocial and other non-pharmacological interventions (e.g., end-of-life care, easy language, aromatherapy, cold/heat application for pain, music therapy) or treated with adequate pharmacological approach when appropriate (e.g., dementia medications, pain relievers). |
| Category:  Environment | | The environment supports the patient in orientation, activation, and independence and provides safety, creates familiarity and calm. |
| Subcategory:  *Orientation* | | Temporal, local and situational orientation of the patient is promoted by the environment. |
|  | **Operationalization:** |  |
|  | Temporal orientation aids | To promote temporal orientation during the hospital stay, various temporal orientation aids are used such as clearly visible and legible clocks and calendars in the patient room and on the ward, circadian lighting and windows for sufficient daylight. |
|  | Local & situational guidance | To promote local orientation, different guides are used such as color coding (e.g., personal area in patient room, ward colors, restroom doors), color contrasts (e.g., light switch, toilet, handrail, plates and cups), guidance systems (e.g., using visual markers with pictures, colors, hallway signage, or light guidance systems) and signs with words, pictograms, pictures and/or photos to mark the patient room or functions of the room (e.g., bathroom, toilet, common room), tactile stimuli (e.g., on handrails, walls) and legibility of ground plan.  Local orientation guidance such as color contrasts of toilets or pictures (e.g., function of the room) also provide situational orientation.  Additionally, information boards with information on daily structure and scheduling (e.g. treatment and examination times, meals), names or photos of responsible staff and details of place of stay (e.g., hospital, ward, room) are used to promote local and situational orientation.  Situational orientation is also promoted by other subcategories of the environment such as the subcategory familiar. |
| Subcategory:  *Activation* | | The environment promotes patient activation in the form of movement, social interaction, and independent engagement with the environment. |
|  | **Operationalization:** |  |
|  | Spaces | Space for social interaction, movement and activities in the form of seating areas, common area/room, sufficient room size and/or hallway design. |
|  | Activity items | The environment is designed with different items to occupy and stimulate the patient, such as books, newspapers, drinks and food, art, pictures, television and/or radio. |
| Subcategory:  *Familiar* | | The environment creates familiarity for the patient through familiar persons, personal items, homely design, and customizable interiors. |
|  | **Operationalization:** |  |
|  | Familiar person | To make the environment familiar to the patient, persons they know are located within the patient’s environment, e.g., relatives or staff. |
|  | Personal items | The environment is decorated, if possible, with personal items, e.g., photos, pillows, own clothes and favorite cup. |
|  | Homelike design | The environment is homelike, designed by providing premises such as patient rooms, common rooms, hallways with homelike furniture, pictures, wall colors, wallpaper, pleasant lighting, and a less functional design (e.g., medical equipment is removed or covered). |
|  | Customized interior | Interior design is customizable (e.g., lighting is customizable, pictures can be replaced, furniture can be rearranged). |
| Subcategory:  *Calm* | | The environment conveys calm to the patient by reducing environmental stimuli and providing comfort. |
|  | **Operationalization:** |  |
|  | Environmental stimulus | Environmental stimuli are reduced in the form of small units (i.e., few patients), separate areas (e.g., emergency room, (partially) segregated wards, movable walls, retreats), a reduction in noise (e.g., sound attenuation in hallways, light alarms), avoidance of shadowing and reflection (e.g., by certain floor coverings, indirect light) simplicity of environment (e.g., few medical devices). |
|  | Comforts | The environment offers comforts in the form of comfortable resting options, adjustment of light and temperature, and if possible, a balcony or terrace. |
| Subcategory:  *Independence and safety* | | The environment promotes the patient's independence while providing safety through measures that enable or limit access and various aids. |
|  | **Operationalization:** |  |
|  | Access | The environment is designed differently to enable or limit access.  To enable independent access to and use of the premises, aspects of barrier-free design are considered. The environment is designed with handrails, adapted floor coverings (e.g., non-slip and pattern-free), no tripping hazards, adequate lighting, seating for resting or booster seats.  There is limited access to hazards (e.g., stairs) and exits to reduce risks of injury or leaving the ward or hospital unnoticed and getting lost. For this purpose, some measures in the environment design are described, such as the design of inconspicuous exits, hiding the doors by design, having only tiltable windows. |
|  | Aids | Aids that provide safety for the patients and promote their independence such as special beds, sensory mats, automatic lighting systems and tracking systems are used. |
| Category:  Valuing relatives | | Valuing relatives (relatives are defined here as persons who are close to the patient with dementia, i.e., they can be family members but also friends and neighbors) is characterized by a welcoming culture for relatives (always welcome), recognition of relatives as partners and experts, their involvement during the patient’s hospital stay, and taking care of them. |
| Subcategory:  *Always welcome* | | Relatives are always welcome in the hospital. They can be with the patient at any time around the clock. The welcoming culture is also reflected in the hospital’s structures and services. |
|  | **Operationalization:** |  |
|  | Visiting hours | Visiting hours are flexible, and there are no restrictions so that relatives can be with patients as long and as often as they wish. |
|  | Rooming-in | Rooming-in is offered for the relatives. They can stay around the clock and have a bed in the patient’s room as well as food and drinks. |
|  | Room & interior | Perspectives of relatives are considered when designing the environment, e.g., in form of retreat areas for relatives (inside and outside the patient’s room), a room for conversations, as well as seating options in waiting areas and examination rooms. |
| Subcategory:  *Recognition* | | Recognizing relatives as experts due to their experience and as partners in the patient’s care. |
|  | **Operationalization:** |  |
|  | As expert by experience | Recognized as experts; consider and value their experience and knowledge. Relatives are experts in the patient's living environment, personal everyday life, and/or care routines. They have a comprehensive knowledge of the patient with dementia (needs, habits, etc.) and could provide insights, for example how to response to a specific behavior (e.g., agitation) and possible triggers (e.g., loud noises). They are listened to, and their views are considered. |
|  | As partner | Relatives are seen as partners in the care process and therefore as a part of the care team. They often support care during the hospital stay and make a significant contribution to discharge management and continuity of care after hospitalization. In this context they should be considered partners in care with whom collaboration is required. |
| Subcategory:  *Involvement* | | Relatives are enabled to be involved during the patient’s hospital stay in different ways related to information (receiving and providing information), mediation between patient and hospital staff, care (active and passive) and decision-making. Involvement of relatives is supported by the welcoming culture. The degree of involvement considers the patient’s wishes and the wishes, burdens and capabilities of the relatives. |
|  | **Operationalization:** |  |
|  | Information | Relatives are informed about the patient's condition, (planned) procedures, medical concerns, possible complications, disease and symptoms, medical treatment, treatment plan, ward routine and about preparation for hospitalization and discharge. On the other hand, they are an important source in providing hospital staff with information about the patient, which is important for treatment, care, and diagnosis (e.g., symptoms, medical history, or information related to knowing the person). |
|  | Mediation | Relatives mediate, if needed, between the patient with dementia and the hospital staff. They assist with communication (e.g., compensate for communication barriers), assist in interpreting patient behaviors and make information and decisions understandable for the patient. |
|  | Care | Relatives may be actively and/or passively involved in care.  Actively involved in the care (e.g., personal hygiene, serving meals, monitoring the patient and structuring the day).  Passively: Relatives are allowed to be present during the patient's care, examinations and medical conversations. They may accompany the patient in the ambulance, in the emergency room and until just before the surgery. They are always welcome and are not routinely asked to leave the room. |
|  | Decisions | Involvement of relatives in decision-making processes (e.g., the necessity of hospital admission, their involvement during hospitalization, choice and control in decisions affecting care and treatment (together with the patient) and discharge planning). |
| Subcategory:  *Taking care* | | Relatives are also taken care of by recognizing and considering their needs, as well as their burden, and offering them tailored support. |
|  | **Operationalization:** |  |
|  | Needs | Identify (e.g., by assessments)/perceive and consider the needs and burdens of relatives. |
|  | Support | Relatives receive support in terms of post-acute care (e.g., discharge planning, applying for support services, home modification, organizing home care/nursing home placement, support services), knowledge about the disease (e.g., delirium, dementia, BPSD and symptoms), carrying out nursing (e.g., nursing training), self-care (e.g., psychological counseling, self-help groups, balance between involvement and taking a break or time-out). Support is provided by means of trainings, counseling, information, assistance in applying for services and self-help groups. |
| Category:  Knowledge and expertise | | The complex care of patients with dementia in the hospital requires different knowledge and expertise related to dementia and various professions and disciplines within the hospital. |
| Subcategory:  *Dementia-specific* | | Dementia-specific knowledge and expertise is available at different levels. All staff have a basic knowledge of dementia. In addition, there are dementia or geriatric experts who can be involved in the care of patients with dementia and support the staff. |
|  | **Operationalization:** |  |
|  | Basic knowledge | Basic knowledge of dementia is available among all hospital staff. However, different dementia specific knowledge is needed according to the involvement of the staff in the care of patients with dementia.  Staff not involved in direct care, such as cleaners, porters, and secretaries, need a basic knowledge of the disease related to their field of activity (e.g., communication, symptoms of dementia).  Staff involved in direct care, such as nurses, physicians, therapists and case managers, need more comprehensive knowledge related to dementia and care in the context of dementia (e.g., assessments, psychosocial interventions, environment, and autonomy). |
|  | Experts | Dementia or geriatric experts (either individuals or teams), are available (at the ward or hospital level) and support the care of patients with dementia in a variety of ways.  They may be consulted for direct involvement in care (e.g., diagnostics, treatment, recommendation of measures) or special tasks in care (e.g., handling behavior that challenges, de-escalation, patient/relatives counseling) and/or they can support the staff (e.g., by collegial counseling, moderating case conferences, providing education and/or skills training in practice). |
| Subcategory:  *Multiprofessional* | | Multiprofessional knowledge and expertise is available for the care of patients with dementia. Therefore, professionals from diverse disciplines are involved in care and different ways of working together are used to bundle the different expertise and knowledge and enable a change of perspective and thinking, whereby the care can be applied in a more holistic manner. |
|  | **Operationalization:** |  |
|  | Involvement | Staff from various professional groups and disciplines are involved in the care of patients with dementia, such as different specialists, geriatric care experts, therapists (e.g., occupational, speech, geriatric, physiotherapists), psychologists, pharmacists, delirium experts and case managers/social workers. |
|  | Working together | The care of patients with dementia takes place in a multiprofessional collaboration. This means that the care of patients with dementia is jointly designed by several professionals in terms of goals, treatment, and discharge planning. Working together can happen in different forms: multiprofessional teams, joint case conferences, joint meetings or networks within the hospital. |
