# Supplementary material for: Characteristics of dementia-friendly hospitals: an integrative review
Source: BMC Geriatr. 2022 May 31;22:468. doi: 10.1186/s12877-022-03103-6 (PMC9158310; doi:10.1186/s12877-022-03103-6)
Supplement: Supplementary file 5 — Additional file 5. Overview of characteristics in each description. [file 12877_2022_3103_MOESM5_ESM.docx]

**Additional file 5:** *Overview of characteristics in each description*

| **Characteristic** | **Research** | | | **Recommendations** | | | | | | **Practice projects** | | | | | | | |
| --- | --- | --- | --- | --- | --- | --- | --- | --- | --- | --- | --- | --- | --- | --- | --- | --- | --- |
|  | [44] | [42] | [43] | [37] | [41] | [40] | [39] | [38] | [36] | [34] | [35] | [28] | [30] | [29] | [31] | [32] | [33] |
| **Continuity** | | | | | | | | | | | | | | | | | |
| Staff |  |  |  | ***x*** | ***x*** | ***x*** |  | ***x*** |  | ***x*** | ***x*** | ***x*** | ***x*** | ***x*** | ***x*** |  | ***x*** |
| Location | ***x*** |  |  | ***x*** | ***x*** | ***x*** | ***x*** | ***x*** | ***x*** |  | ***x*** | ***x*** | ***x*** | ***x*** |  |  | ***x*** |
| Daily structure | ***x*** |  |  | ***x*** | ***x*** | ***x*** |  | ***x*** | ***x*** |  | ***x*** | ***x*** | ***x*** | ***x*** | ***x*** |  | ***x*** |
| Companionship | ***x*** | ***x*** |  | ***x*** | ***x*** | ***x*** |  | ***x*** | ***x*** | ***x*** | ***x*** | ***x*** | ***x*** | ***x*** | ***x*** | ***x*** | ***x*** |
| Being informed | ***x*** | ***x*** |  | ***x*** | ***x*** | ***x*** | ***x*** | ***x*** | ***x*** |  | ***x*** |  |  | ***x*** |  | ***x*** | ***x*** |
| Planning in advance | ***x*** |  | ***x*** | ***x*** | ***x*** | ***x*** | ***x*** | ***x*** | ***x*** | ***x*** | ***x*** | ***x*** | ***x*** | ***x*** |  | ***x*** | ***x*** |
| Crossing sector boundaries | ***x*** |  | ***x*** | ***x*** | ***x*** |  | ***x*** | ***x*** | ***x*** | ***x*** |  | ***x*** |  | ***x*** | ***x*** |  | ***x*** |
| **Person-centeredness** | | | | | | | | | | | | | | | | | |
| Knowing the person | ***x*** | ***x*** |  | ***x*** | ***x*** | ***x*** | ***x*** | ***x*** | ***x*** |  | ***x*** | ***x*** | ***x*** | ***x*** | ***x*** | ***x*** | ***x*** |
| Attitude toward the person |  | ***x*** | ***x*** | ***x*** | ***x*** |  | ***x*** | ***x*** |  |  | ***x*** | ***x*** |  | ***x*** |  | ***x*** | ***x*** |
| Caring for the person | ***x*** | ***x*** |  | ***x*** | ***x*** | ***x*** | ***x*** | ***x*** | ***x*** | ***x*** |  | ***x*** | ***x*** | ***x*** | ***x*** | ***x*** | ***x*** |
| **Consideration of phenomena within dementia*** | | | | | | | | | | | | | | | | | |
| What? Phenomena |  |  |  |  |  |  |  |  |  |  |  |  |  |  |  |  |  |
| Dementia-specific symptoms | ***x*** |  |  | ***x*** | ***x*** | ***x*** | ***x*** | ***x*** | ***x*** | ***x*** | ***x*** | ***x*** | ***x*** | ***x*** | ***x*** | ***x*** | ***x*** |
| Other (care) phenomena & risks | ***x*** |  |  | ***x*** | ***x*** |  | ***x*** | ***x*** | ***x*** | ***x*** | ***x*** | ***x*** | ***x*** | ***x*** |  | ***x*** | ***x*** |
| How? Methods |  |  |  |  |  |  |  |  |  |  |  |  |  |  |  |  |  |
| Identification & diagnostics | ***x*** |  |  | ***x*** | ***x*** | ***x*** | ***x*** | ***x*** | ***x*** | ***x*** | ***x*** | ***x*** | ***x*** | ***x*** |  | ***x*** | ***x*** |
| Prevention, treatment & care intervention | ***x*** |  |  |  | ***x*** | ***x*** | ***x*** | ***x*** | ***x*** | ***x*** | ***x*** | ***x*** | ***x*** | ***x*** | ***x*** | ***x*** | ***x*** |
| **Environment** |  |  |  |  |  |  |  |  |  |  |  |  |  |  |  |  |  |
| Orientation | ***x*** | ***x*** |  | ***x*** | ***x*** | ***x*** | ***x*** | ***x*** | ***x*** | ***x*** | ***x*** | ***x*** | ***x*** | ***x*** |  | ***x*** | ***x*** |
| Activation | ***x*** |  |  | ***x*** | ***x*** | ***x*** | ***x*** | ***x*** | ***x*** |  | ***x*** | ***x*** | ***x*** | ***x*** | ***x*** | ***x*** |  |
| Familiar |  | ***x*** |  | ***x*** | ***x*** | ***x*** |  | ***x*** | ***x*** |  | ***x*** |  | ***x*** | ***x*** |  | ***x*** | ***x*** |
| Calm | ***x*** | ***x*** |  | ***x*** | ***x*** | ***x*** | ***x*** | ***x*** | ***x*** |  | ***x*** | ***x*** | ***x*** | ***x*** | ***x*** |  | ***x*** |
| Independence & safety | ***x*** | ***x*** |  | ***x*** | ***x*** | ***x*** |  | ***x*** | ***x*** | ***x*** | ***x*** | ***x*** | ***x*** | ***x*** | ***x*** | ***x*** |  |
| **Valuing relatives** |  |  |  |  |  |  |  |  |  |  |  |  |  |  |  |  |  |
| Always welcome | ***x*** |  |  | ***x*** | ***x*** | ***x*** | ***x*** | ***x*** | ***x*** |  | ***x*** | ***x*** | ***x*** | ***x*** |  | ***x*** |  |
| Recognition | ***x*** |  |  | ***x*** | ***x*** | ***x*** | ***x*** | ***x*** | ***x*** | ***x*** |  | ***x*** |  | ***x*** | ***x*** | ***x*** | ***x*** |
| Involvement | ***x*** | ***x*** |  | ***x*** | ***x*** | ***x*** | ***x*** | ***x*** | ***x*** | ***x*** | ***x*** | ***x*** | ***x*** | ***x*** | ***x*** | ***x*** | ***x*** |
| Taking care | ***x*** | ***x*** | ***x*** | ***x*** | ***x*** | ***x*** | ***x*** | ***x*** | ***x*** | ***x*** | ***x*** | ***x*** | ***x*** | ***x*** | ***x*** |  | ***x*** |
| **Knowledge & expertise** |  |  |  |  |  |  |  |  |  |  |  |  |  |  |  |  |  |
| Dementia-specific | ***x*** | ***x*** |  | ***x*** | ***x*** | ***x*** | ***x*** | ***x*** | ***x*** | ***x*** | ***x*** | ***x*** | ***x*** | ***x*** | ***x*** | ***x*** | ***x*** |
| Multiprofessional | ***x*** |  |  | ***x*** | ***x*** | ***x*** | ***x*** | ***x*** | ***x*** | ***x*** | ***x*** | ***x*** | ***x*** | ***x*** | ***x*** | ***x*** | ***x*** |

*Characteristic is presented with two sublevels due to its special structure

Note: For clarity, only the primary literature is listed, including all publications on the description of DFHs (see Table 2).
